# Exonuclease III-Regulated Target Cyclic Amplification-Based Single Nucleotide Polymorphism Detection Using Ultrathin Ternary Chalcogenide Nanosheets

**DOI:** 10.3389/fchem.2019.00844

**Published:** 2019-12-06

**Authors:** Yanling Hu, Chaoliang Tan, Xin Lin, Zhuangchai Lai, Xiao Zhang, Qipeng Lu, Ning Feng, Dongliang Yang, Lixing Weng

**Affiliations:** ^1^School of Electrical and Control Engineering, Nanjing Polytechnic Institute, Nanjing, China; ^2^Jiangsu Key Laboratory for Biosensors, Institute of Advanced Materials (IAM), Nanjing University of Posts and Telecommunications, Nanjing, China; ^3^School of Materials Science and Engineering, Nanyang Technological University, Singapore, Singapore; ^4^School of Ophthalmology and Optometry, Eye Hospital, Wenzhou Medical University, Wenzhou, China; ^5^School of Physical and Mathematical Sciences, Nanjing Tech University (NanjingTech), Nanjing, China

**Keywords:** ternary chalcogenide nanosheets, single nucleotide polymorphisms, two-dimensional nanomaterials, fluorescent detection, sensor

## Abstract

Herein, we report that the ternary chalcogenide nanosheet exhibits different affinity toward oligonucleotides with different lengths and efficiently quenches the fluorescence of dye-labeled DNA probes. Based on these findings, as a proof-of-concept application, the ternary chalcogenide nanosheet is used as a target cyclic amplification biosensor, showing high specificity in discriminating single-base mismatch. This simple strategy is fast and sensitive for the single nucleotide polymorphism detection. Ultralow detection limit of unlabeled target (250 fM) and high discrimination ratio (5%) in the mixture of perfect match (mutant-type) and single-base mismatch (wild-type) target are achieved. This sensing method is extensively compatible for the single nucleotide polymorphism detection in clinical samples, making it a promising tool for the mutation-based clinical diagnostic and genomic research.

## Introduction

Single nucleotide polymorphisms (SNPs) are agents of various diseases such as cancers, Alzheimer disease, and diabetes (Martin et al., 2000; Syvanen, 2001; Unoki et al., 2008). Therefore, methods to discriminate SNPs are of significant importance as the first step for the disease prediction and clinical diagnosis. In the past two decades, several approaches have been developed for the SNP detection, such as ligation chain reaction, molecule beacons, and surface-enhanced Raman scattering (Chen et al., 2013, 2019; Li et al., 2019; Li Y. et al., 2019). However, the small thermodynamic energy difference between the perfect match and single-base mismatch makes it difficult to achieve both good sensitivity and high SNP discrimination (Zhang et al., 2013; Wu et al., 2015). Although many efforts have been made to develop analysis technologies and detection platforms for improving the sensitivity and specificity, such as polymerase chain reaction (PCR) technique and enzyme-assisted methods, sophisticated biological systems are designed to distinguish such mismatch due to allele-specific oligonucleotides hybridization and enzyme recognition (Mhlanga and Malmberg, 2001; Doerks et al., 2002). Especially, high cost, complicated primer design, and specific enzyme recognition sites restrict their practical applications (Mhlanga and Malmberg, 2001; Nazarenko et al., 2002; Li et al., 2008; Gerasimova and Kolpashchikov, 2014; Chang et al., 2015; Huang et al., 2019). Over the past decades, nanomaterials have been greatly explored as biosensing platforms (Yang et al., 2016; Cai et al., 2018; Qiu et al., 2019; Zeng et al., 2019). As a new class of nanomaterials, single- and few-layered transition metal dichalcogenide (TMD) nanosheets have attracted tremendous attention (Chhowalla et al., 2013; Tan et al., 2017; Chen et al., 2018). Owing to their unique electronic, optical, chemical properties, and low toxicity (Zhang et al., 2018; Su et al., 2019), TMD nanosheets exhibit great potential in various applications including catalysis (Woods et al., 2016; Tang et al., 2019), electronic devices (Zhu W. et al., 2019), energy storage (Yun et al., 2019), and sensors (Chen et al., 2015; Li et al., 2015; Hu et al., 2017; Xu et al., 2019). Owing to their high-efficiency electron transfer, high surface/volume ratio, and easy dispersibility in water, TMD nanosheets have been chosen as competitive candidates for biosensing (Bolotsky et al., 2019). For example, our group previously reported that the MoS_2_ nanosheet can serve as a platform to construct a fluorescent sensor for detection of DNA and small molecules due to its fluorescence quenching ability and adsorption of dye-labeled single-stranded (ss) DNA (Zhu C. et al., 2013). This strategy has been extended to other TMD nanosheets, such as WS_2_, TaS_2_, and TiS_2_ (Ge et al., 2014; Zhang et al., 2015; Zhu D. et al., 2019). Recently, the ternary chalcogenide nanosheet, i.e., Ta_2_NiS_5_, also exhibited the similar ability for DNA detection and show better performance than that of MoS_2_ (Tan et al., 2015). Therefore, efforts can be made in the exploration of applications of Ta_2_NiS_5_ nanosheets with good sensitivity and specificity, so that the sensing platform can be used in clinical diagnose especially genetic-variation-related diseases.

Here, the ternary chalcogenide nanosheet, i.e., Ta_2_NiS_5_, is used as a target cyclic amplification biosensor for the SNP detection, showing high sensitivity and good specificity. A dye-labeled DNA is used as probe (P) for the detection of mutant-type target (MT). Wild-type target (WT) is used to evaluate the SNP discrimination.

## Materials and Methods

### Materials

DNA sequences were synthesized and purified by Sangon Biotechnology Co., Ltd. (Shanghai, China). Exonuclease III (Exo III), NEBuffer 1, and loading buffer were purchased from New England Biolabs (Singapore). DNA ladder was purchased from Takara Biotechnology Co. Ltd. (Dalian, China). Tris–acetate–ethylenediaminetetraacetic acid buffer (50 ×) was purchased from Axil Scientific Pte Ltd. Acrylamide/bis mixed solution [30% (*w*/*v*] (29:1) was purchased from Nacalai Tesque, Inc. Ammonium persulfate and tetramethylethylenediamine were purchased from Biorad. All these chemicals were used without further purification. The Milli-Q water was obtained through a Milli-Q system (Millipore) and was used in all the experiments.

### Preparation of Single-Layer Ta_2_NIS_5_ Nanosheets

The single-layer Ta_2_NiS_5_ nanosheets were synthesized based on the lithium-intercalation method developed by our group (Zeng et al., 2011). The obtained suspension was then centrifuged and washed with water for four times. The final product was collected for further experiments.

### Characterization

Transmission electron microscopy (TEM) images were taken by a JEOL JEM-2100F transmission electron microscope. Atomic force microscopy (AFM) images were recorded by a Dimension 3100 AFM (Veeco, Fremont, CA, USA) in tapping mode. Fluorescence measurements were performed on a Shimadzu RF-5301 PC fluorophotometer.

### Single-Nucleotide Polymorphism Detection

In a typical hybridization and digestion process, 5 μl of probe (P, 10 μM) was hybridized with a series of MT at increasing concentrations (5 μl, 0–1 μM) premixed with 5 μl of Exo III (2.5 U μl^−1^) in 330 μl of NEBuffer 1 work solution for 30 min at 37°C. Two microliters of Ta_2_NiS_5_ nanosheets (0.125 mg ml^−1^) was added to the mixture and incubated for 5 min. Then, the fluorescence measurements were carried out with the final concentration of MT (0–100 nM). The excitation and emission wavelengths were 590 and 610 nm, respectively.

### Gel Electrophoresis Analysis

Gel electrophoresis analysis was performed using 12% polyacrylamide gel. The electrophoresis was carried out at 84 V for 100 min in 1 × Tris–acetate–ethylenediaminetetraacetic acid with a load of 20 μl of sample containing 2 μl of loading buffer. Then, the gel was stained with ethidium bromide for 10 min. The gel images were obtained by a charge-coupled device under UV lamp illumination.

### Cell Culture, Cellular Extracts Preparation, and Human Genomic Sample Analysis

Lung cancer cell lines (A549) and induced pluripotent stem cells were purchased from the American Type Culture Collection. The cells were cultured in Dulbecco's modified Eagle's medium supplemented with 10% fetal bovine serum, 100 μg ml^−1^ streptomycin, and 100 U ml^−1^ penicillin. All cells were maintained in a humidified incubator at 37°C containing 5% CO_2_.

DNA was extracted from A549 cells and induced pluripotent stem cells with TRIzol following the manufacturer's protocol, respectively. Briefly, approximately 1 × 10^7^ cells were harvested and washed once with phosphate-buffered saline (pH 7.4, containing 137 mM NaCl, 2.7 mM KCl, 10 mM Na_2_HPO_4_, and 2.0 mM KH_2_PO_4_). Then, the total DNA was isolated according to the manufacturer's instruction and quantified using a NanoDrop 1000 Spectrophometer (Thermo Scientific).

Target DNA amplification was performed in 100 μl of reaction mixture with 200 μM deoxyribonucleoside triphosphates, 1.5 mM MgCl_2_, 1 μM forward and reverse primers, 0.02 U μl^−1^ KOD Enzyme, 10 μl buffer (10 × buffer for KOD Hot Start DNA Polymerase) and genomic DNA (lung cancer DNA, 1.1 ng; normal DNA, 1.2 ng). The sequences of primers used in the experiment are listed in Table S1. After an initial denaturation at 95°C for 1 min, the amplification was achieved by 29 cycles of thermal cycling at 95°C for 20 s, 52°C for 10 s, and 70°C for 1 min to get 359 bp PCR products. The PCR products were used for subsequent experiments after purification with E.Z.N.A.^®^ Cycle Pure Kit (Omega Bio-Tek Inc., Doraville, GA, USA) following the manufacturer's protocol. DNA sequencing was carried out by DNA Sequencing Facility (Institute of Molecular and Cell Biology, Singapore).

## Results and Discussion

### Sensing Mechanism

**Scheme 1:**
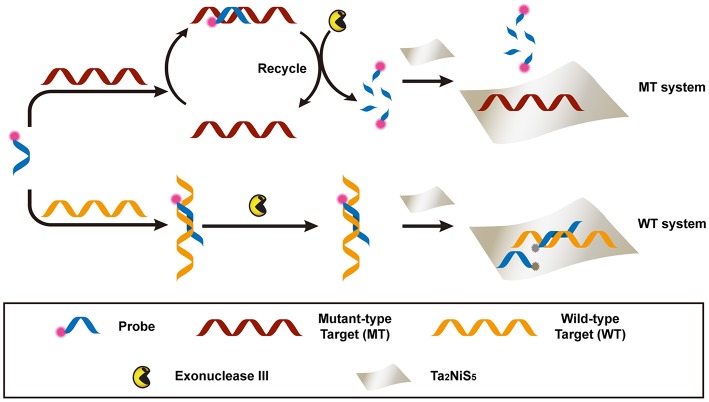
Schematic illustration of the Ta_2_NiS_5_ nanosheet-based fluorescent sensor for the single nucleotide polymorphism (SNP) detection.

The proposed detection process is shown in Scheme 1. Briefly, the dye-labeled DNA probe (P) and MT were incubated together to form perfectly matched double-stranded (ds) DNA, used as the substrate of exonuclease III (Exo III) digestion. For the perfectly match system, Exo III could digest one strand of a duplex from the recessed 3′ termini, so P was cleaved to shorter oligonucleotides. Meanwhile, MT was released and able to hybridize with other P in solution. This facilitated a new round of hybridization, digestion, and release process. Owing to the weak affinity between the Ta_2_NiS_5_ nanosheet and short oligonucleotides, the fluorescence of P would partially remain (Wu et al., 2011). In addition, the recycled process prompted an amplification of fluorescence signal. This is in contrast to the WT system, in which WT could form single-base mismatched duplex with P at 3′ termini. The mismatched base prevented Exo III from digesting P, resulting in the fluorescence quenching after addition of Ta_2_NiS_5_ nanosheets. The DNA sequences used in this experiment are listed in Table S1.

### Characterization of Ta_2_NIS_5_ Nanosheet

**Figure 1:**
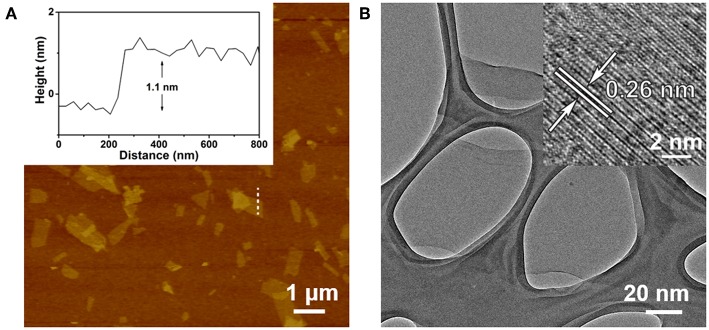
**(A)** Atomic force microscopy (AFM) height image of Ta_2_NiS_5_ nanosheets with average height of 1.1 nm. **(B)** Transmission electron microscopy (TEM) image of Ta_2_NiS_5_ nanosheets. Inset: the corresponding high-resolution TEM (HRTEM) image.

The Ta_2_NiS_5_ nanosheet was prepared through the electrochemical lithium-intercalation method according to our previous method (Zeng et al., 2011; Tan et al., 2015). AFM measurement revealed that the average thickness of Ta_2_NiS_5_ nanosheet is ~1.1 nm, indicating that the Ta_2_NiS_5_ nanosheet is single layer in thickness (Figure 1A) (Tan et al., 2015). The TEM and high-resolution TEM images of the Ta_2_NiS_5_ nanosheet are shown in Figure 1B. The measured lattice spacing from the high-resolution TEM is 0.26 nm, corresponding to the (006) planes of Ta_2_NiS_5_ (Tan et al., 2015). Previous studies indicate that the oligonucleotides can be absorbed to two-dimensional nanomaterials, and the fluorescence of dye-labeled oligonucleotides can be quenched due to the photo-induced electron transfer happening between the aromatic fluorescent dyes and two-dimensional nanomaterials (Ramakrishna Matte et al., 2011). After preparation of Ta_2_NiS_5_ nanosheets, their fluorescence quenching ability toward ssDNA with different lengths was studied. As shown in Figure S1, the Ta_2_NiS_5_ nanosheet exhibits the fluorescence quenching efficiency up to 50, 85, 99, and 99% toward ssDNA with 5, 10, 15, and 20 bases, demonstrating that for ssDNA with <15 bases, the affinity between ssDNA and Ta_2_NiS_5_ nanosheet increases along with the length of ssDNA, resulting in higher fluorescence quenching efficiency.

### Feasibility Analysis

**Figure 2:**
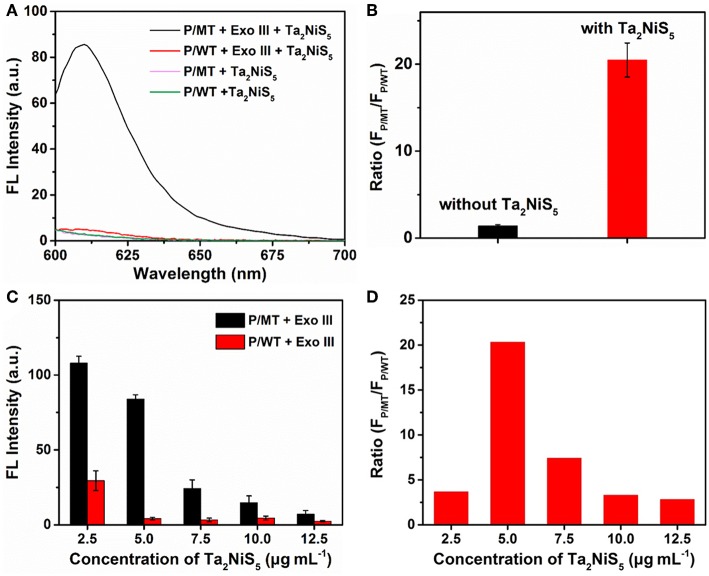
**(A)** Fluorescence spectra of P/MT + Exo III + Ta_2_NiS_5_ (black), P/WT + Exo III + Ta_2_NiS_5_ (red), P/MT + Ta_2_NiS_5_ (pink), and P/WT + Ta_2_NiS_5_ (green). **(B)** The fluorescence intensity ratio (FP/MT/FP/WT) at 610 nm for P/MT + Exo III and P/WT + Exo III in the absence (black) and presence (red) of Ta_2_NiS_5_ nanosheets. **(C)** Fluorescence intensity of P/MT + Exo III (black) and P/WT + Exo III (red) in the presence of Ta_2_NiS_5_ nanosheets with different final concentrations of 2.5, 5.0, 7.5, 10.0, and 12.5 μg ml^−1^ (P = 1 μM; MT = 100 nM; WT = 100 nM; Exo III = 0.25 U μl^−1^). **(D)** The fluorescence intensity ratio (FP/MT/FP/WT) at 610 nm in the presence of Ta2NiS5 nanosheets with different final concentrations of 2.5, 5.0, 7.5, 10.0, and 12.5 μg ml^−1^ (P = 1 μM; MT = 100 nM; WT = 100 nM; Exo III = 0.25 U μl^−1)^. The excitation wavelength is 590 nm.

To investigate the feasibility of our designed method for the single-base mismatch discrimination, fluorescent spectra of P were carried out under different conditions of assays, as shown in Figure 2A. When P (1 μM) was incubated with MT (100 nM) with the addition of Ta_2_NiS_5_ nanosheet (5.0 μg ml^−1^), only weak fluorescence can be observed [pink curve (P/MT + Ta_2_NiS_5_) in Figure 2A]. The fluorescence spectrum of P/WT in the presence of Ta_2_NiS_5_ also showed weak fluorescence intensity [green curve (P/WT + Ta_2_NiS_5_) in Figure 2A]. When Exo III was employed in the aforementioned system, the fluorescence intensity retained for the perfect match system of P and MT [black curve (P/MT + Exo III + Ta_2_NiS_5_) in Figure 2A], indicating that P was digested by Exo III. As a result of the weak interaction between fluorophore and Ta_2_NiS_5_ nanosheet, the fluorescence signal could be partially recovered. However, for the single-base mismatch system, no significant fluorescence increase was observed [red curve (P/WT + Exo III + Ta_2_NiS_5_) in Figure 2A], suggesting that the fluorophore was adsorbed on the surface of Ta_2_NiS_5_ nanosheet. Moreover, the discrimination ratio of P/MT + Exo III to P/WT + Exo III, referred to as *F*_P/MT_/*F*_P/WT_, where *F*_P/MT_ and *F*_P/WT_ are the fluorescence signals of MT and WT systems, respectively, was 1.5 and 20.5 in the absence and presence of Ta_2_NiS_5_ nanosheet, respectively (Figure 2B). It suggests that the introduction of Ta_2_NiS_5_ nanosheets results in the effective discrimination of SNP. The digestion process has also been confirmed through the gel electrophoresis (Figure S2). These results demonstrated that the single-base mismatch at 3′ terminus can weaken the digestion ability of Exo III (Figure S3), and the Ta_2_NiS_5_ nanosheet plays an important role in improving the single-base mismatch discrimination.

### Optimization of Detection Conditions

To achieve better assay performance, we optimized the digestion and sensing conditions, including the concentration and digestion time of Exo III in the digestion process and the amount of Ta_2_NiS_5_ nanosheets. As shown in Figure S4, after using Exo III digestion for 30 min, fluorescence intensity of P/MT almost reaches the maximum value. Therefore, the reaction time of biosensor was chosen at 30 min. To optimize the concentration of Exo III, *F*_P/MT_/*F*_P/WT_ was used as the criterion. The dependence of *F*_P/MT_/*F*_P/WT_ on the concentration of Exo III is illustrated in Figure S5, showing maximum value at 0.25 U μl^−1^ of Exo III. Therefore, 0.25 U μl^−1^ was chosen for the concentration of Exo III and used throughout the subsequent assays. Besides the concentration of Exo III, the concentration of Ta_2_NiS_5_ nanosheets should also affect the fluorescence response. As shown in Figure 2C, the fluorescence intensity changes of P/MT + Exo III and P/WT + Exo III were carried out in the presence of different concentrations of Ta_2_NiS_5_ nanosheets. Both *F*_P/MT_ and *F*_P/WT_ decreased along with the increasing concentration in Ta_2_NiS_5_ nanosheet, while *F*_P/MT_ was always higher than *F*_P/WT_. Figure 2D displayed the dependence of *F*_P/MT_/*F*_P/WT_ on the concentration of Ta_2_NiS_5_ nanosheets. *F*_P/MT_/*F*_P/WT_ increased with an increasing concentration of Ta_2_NiS_5_ nanosheets with maximum value at the final Ta_2_NiS_5_ nanosheet concentration of 5.0 μg ml^−1^. Then, *F*_P/MT_/*F*_P/WT_ decreased with further increasing concentration of Ta_2_NiS_5_ nanosheets, resulting from the adsorption of short dye-labeled oligonucleotides with excess Ta_2_NiS_5_ nanosheets. Thus, the concentration of Ta_2_NiS_5_ nanosheet was optimized to be 5.0 μg ml^−1^ for further experiments.

### Sensitivity and Specificity Analysis

**Figure 3:**
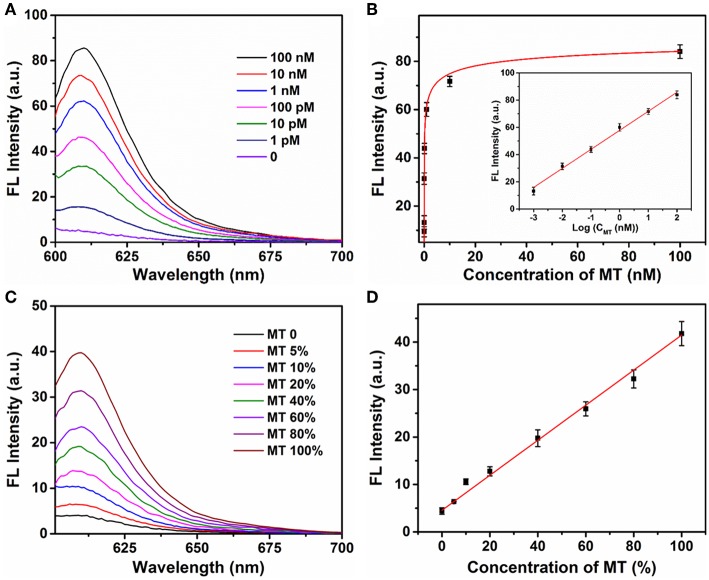
**(A)** The fluorescence spectra of P (1 μM) in the presence of different concentrations of mutant-type target (0, 0.001, 0.01, 0.1, 1, 10, and 100 nM) and Exo III (0.25 U μl^−1^) with addition of Ta_2_NiS_5_ nanosheets (5.0 μg ml^−1^). **(B)** Relationship between fluorescence intensity at 610 nm and the concentrations of mutant-type target. Inset: Calibration curve for detection of mutant-type target. **(C)** Fluorescence spectra of different percentage of mutant-type target in mixed DNA samples (MT/(MT + WT) was 0, 5, 10, 20, 40, 60, 80, and 100%). **(D)** Fluorescence intensity at 610 nm as a function of allele frequency. The total concentration of the mutant and wild-type target is 100 pM. The excitation wavelength is 590 nm.

The sensitivity of the Ta_2_NiS_5_ nanosheet-based biosensor was examined under the optimal conditions. Different concentrations of MT from 0 to 100 nM were incubated with P (1 μM) in the presence of Exo III (0.25 U μl^−1^) for 30 min and then mixed with the Ta_2_NiS_5_ nanosheets (5.0 μg ml^−1^). The fluorescence intensity increases along with the increase in the MT concentration (Figure 3A). As shown in Figure 3B, the fluorescence intensity showed a linear correlation vs. a series of MT concentrations in logarithmic scale in the range from 1 pM to 100 nM. The calibration equation was *Y* = 57.46 + 13.97 lg *X* (*R*^2^ = 0.9952), where *Y* stands for the fluorescence intensity and *X* is the concentration of MT. According to the 3σ rule, the limit of detection was calculated to be 250 fM (Hu et al., 2018). The comparison of Ta_2_NiS_5_ nanosheet-based biosensor with the conventional reported SNP biosensor based on Ti_3_C_2_ nanosheet, GO nanosheet, MoS_2_@Au NPs, and Au NPs is presented in Table S2; the Ta_2_NiS_5_ nanosheet-based exhibits high sensitivity for SNP detection.

Different ratios of MT and WT were mixed and used as DNA samples for the analysis of allele sequence. Detection results were carried out for mixtures with various ratios of MT (0, 5, 10, 20, 40, 60, 80, and 100%). The total concentration of MT and WT was 100 pM. Figure 3C showed the fluorescence spectra of P with a series ratio of MT in the presence of Exo III (0.25 U μl^−1^) and Ta_2_NiS_5_ nanosheet (5.0 μg ml^−1^). The statistic curve of the relationship between fluorescence intensity and the percentage of MT in the tested samples is shown in Figure 3D. The increasing amount of MT in DNA mixtures led to an increase in fluorescence intensity at 610 nm. Moreover, MT, as low as 5%, could be detected. These results indicated that this strategy afforded high specificity as well as good sensitivity for SNP detection due to the quenching ability of Ta_2_NiS_5_ nanosheet and low detection background together with signal amplification of target cyclic amplification reaction. Based on this, the proposed method can be considered a potential candidate for mutant detection in practical cancer research.

The applicability of the proposed single nucleotide discrimination method was further validated by the PCR product of human genomic samples with a point mutant (C > T) in the CHRNA3 gene (rs1051730) (Han et al., 2015). Previous researches confirm that rs1051730 polymorphism have a significant correlation with lung cancer in the East Asia countries (Han et al., 2015). Therefore, rs1051730 polymorphism detection was performed. The 359-bp amplicons were obtained by PCR from four DNA samples (samples 1 and 2: mutant-type samples; samples 3 and 4: wild-type samples). Agarose gel analysis was performed to confirm the PCR amplicons (Figure S6). Then, the PCR amplicons were used for the mutation detection through the proposed method (Figure S7). It was observed that the fluorescence signals of samples 1 and 2 were much higher than those of samples 3 and 4, indicating that samples 1 and 2 were mutant type and could be distinguished from wild-type samples. These results were consistent with the DNA sequence data, indicating the potential application of the developed method for SNP detection of real samples.

## Conclusions

In summary, we reported a ternary chalcogenide nanosheet-based biosensor combined with target cyclic amplification as a mean of SNP detection, with a discrimination of ~20.5. This strategy is based on the high quenching ability of the Ta_2_NiS_5_ nanosheet and different affinity between the Ta_2_NiS_5_ nanosheet and oligonucleotides. The proposed sensing method is a label-free approach for detection of DNA concentrations from 1 pM to 100 nM, and high sensitivity with a detection limit of 250 fM was achieved. Perfectly matched base pairs can be efficiently discriminated from single-base mismatched pairs. Compared with conventional SNP analytical methods such as droplet digital PCR and DNA sequencing, the single-layer Ta_2_NiS_5_ nanosheet-based biosensor displays the advantages of low cost and simplicity. Significantly, this biosensor platform shows better performance compared to previously reported nanomaterial-based SNP sensors (e.g., Ti_3_C_2_ nanosheet and MoS_2_@Au NPs) with broader detection range and a low detection limit. Furthermore, this method shows application potential for multiplexed assays by utilizing different DNA probes with diverse fluorophores. We anticipate that the proposed strategy can be used for clinical diagnostic and genomic research with great potential.

## Data Availability Statement

All datasets generated for this study are included in the article/Supplementary Material.

## Author Contributions

YH, LW, and CT conceived the project. YH, CT, and DY performed the experiments and wrote the manuscript. YH, ZL, XZ, NF, XL, and QL were involved in data analysis. All authors revised the manuscript and approved it for publication.

### Conflict of Interest

The authors declare that the research was conducted in the absence of any commercial or financial relationships that could be construed as a potential conflict of interest.
