# Supplementary material for: Exonuclease III-Regulated Target Cyclic Amplification-Based Single Nucleotide Polymorphism Detection Using Ultrathin Ternary Chalcogenide Nanosheets
Source: Front Chem. 2019 Dec 6;7:844. doi: 10.3389/fchem.2019.00844 (PMC6913186; doi:10.3389/fchem.2019.00844)
Supplement: Supplementary file 1 [file Data_Sheet_1.pdf]

Supplementary Materials

1 Supplementary Table

Table S1. DNA sequences used in the experiments.

Name	Sequences (5' to 3')
Probe (P)	Texas Red-TTGATGTCGTGTTTA
P5	Texas Red-TTGAT
P10	Texas Red-TTGATGTCGT
P20	Texas Red-TTGATGTCGTGTTTATAGCC
Mutant-type target (MT)	AGGCTATAAACACGACATCAAGTAC
Wild-type target (WT)	AGGCTACAAACACGACATCAAGTAC
Primer-Forward	GTGATGTCGGGGTAGATCTCC
Primer-Reverse	GACCAAGTGTTAATGACCGTG

The mutant site (rs1051730 (Han et al., 2015)) is marked in red underline.

2 Supplementary Figures

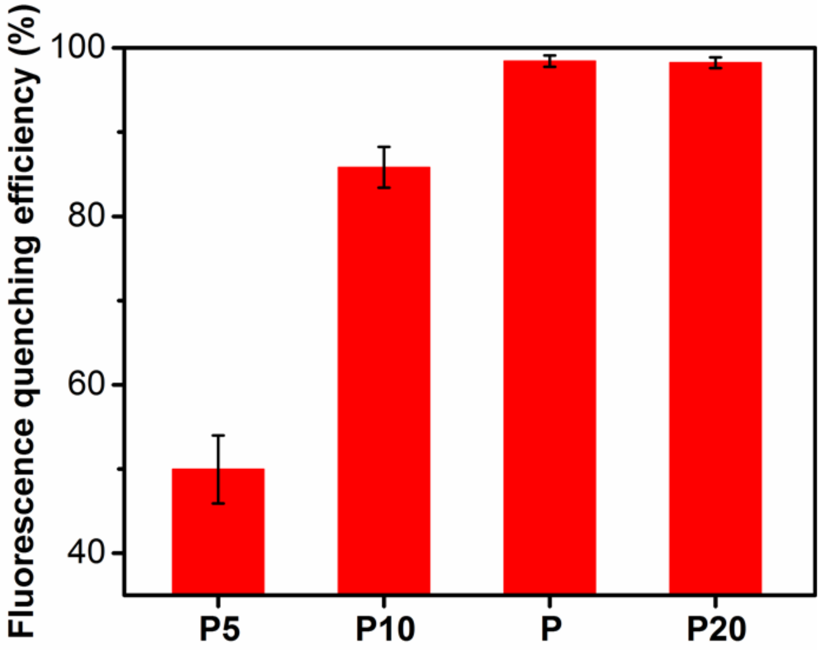

Figure S1. Fluorescence quenching efficiencies ($(F_0$-$F)/F_0$) of single-layer Ta_2NiS_5 nanosheets for single-stranded DNA probes with different lengths. The concentration of Ta_2NiS_5 nanosheets was fixed at 5.0 µg mL^{-1}. P5, P10, P, and P20 denote the probes showed in Table S1.

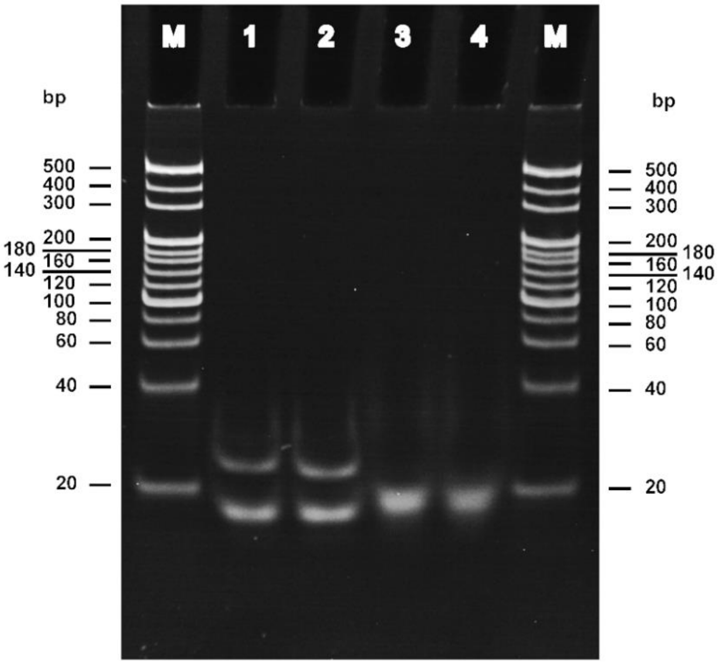

Figure S2. Polyacrylamide gel electrophoresis image for the hybridization of probe DNA and target DNA. Lane M: DNA marker; lane 1: 1 μM P and 100 nM mutant-type target; lane 2: 1 μM P and 100 nM wild-type target; lane 3: 1 μM P and 100 nM mutant-type target with 0.25 U μL^{-1} Exo III; and lane 4: 1 μM P and 100 nM wild-type target with 0.25 U μL^{-1} Exo III.

The digestion process was confirmed by the polyacrylamide gel electrophoresis. As shown in Fig. S3, double-strand DNAs were formed by the hybridization of probe (P) with the mutant-type target (MT) and the wild-type target (WT), respectively, exhibiting two bright bands with almost the same mobility (lanes 1 and 2). Each band in both lane 1 and 2 was accompanied by another band due to the excess P. Upon adding Exo III into the aforementioned system, migration shifts of the bright bands were observed and the bright band in lane 3 showed more shift than that in lane 4, suggesting that P was completely digested in MT system and partially digested in WT system due to the single-base mismatch with WT.

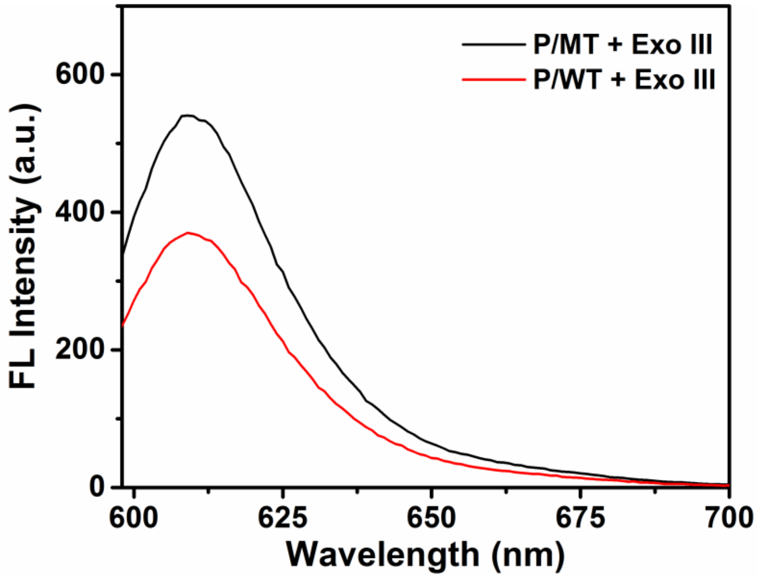

Figure S3. Fluorescence spectra of P/MT + Exo III (black), and P/WT + Exo III (red) in the absence of Ta_2NiS_5 nanosheets (P = 1 μM; MT = 100 nM; WT = 100 nM, Exo III = 0.25 U μL^{-1}). The excitation wavelength is 590 nm.

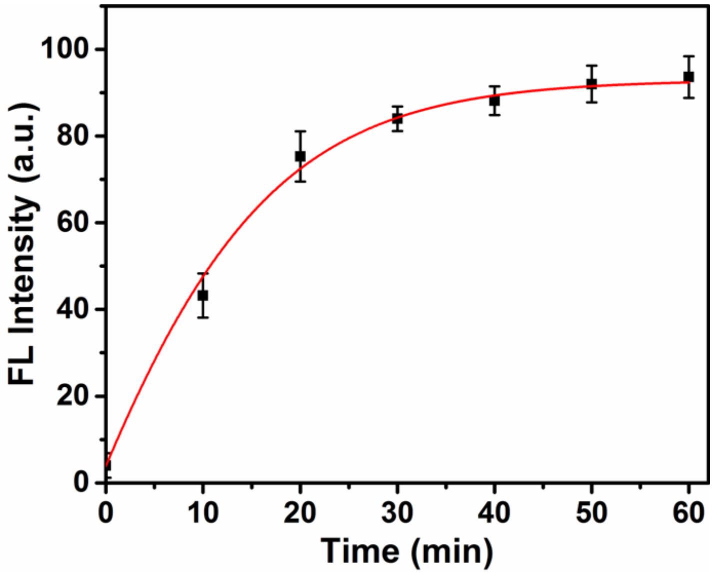

Figure S4. Fluorescence intensity of P/MT after different incubation time with Exo III (0.25 U μL^{-1}) in the presence of Ta_2NiS_5 (5 μg mL^{-1}).

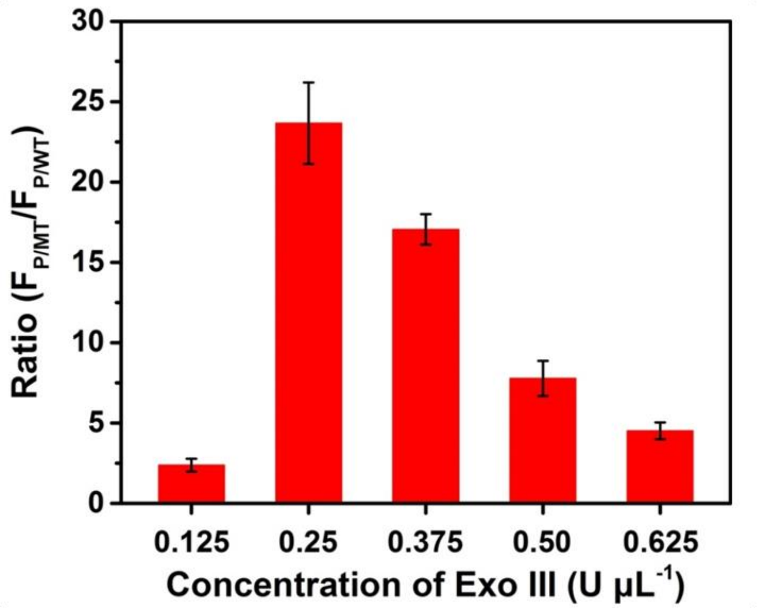

Figure S5. The fluorescence intensity ratio ($F_{P/MT}/F_{P/WT}$) at 610 nm with different concentration of Exo III (0.125, 0.25, 0.375, 0.50 and 1.0 U μL^{-1}) in the presence of Ta_2NiS_5 nanosheets (5.0 μg mL^{-1}). The concentration of P, MT, and WT are 1 μM, 100 nM, and 100 nM, respectively. The volume of solution is fixed at 50 μL. The excitation wavelength is 590 nm.

Table 2 Comparison of SNP biosensors.

**Nanomaterial platform**	**Detection type**	**LOD**	**Linear range**	**Reference**
Ta_2NiS_5 nanosheet	Fluorescence	250 fM	1 pM-100 nM	This work
Ti_3C_2 nanosheet	Electrochemiluminescence	1 nM	NA	(Fang et al., 2018)
MoS_2@Au NPs	Fluorescence	1 nM	0-10 nM	(Yan et al., 2019)
Au NPs	Colorimetry	6.1 nM	50 nM-200 nM	(Xing et al., 2019)
SWNTs	Fluorescence	60 nM	100 nM- 1 μM	(Xu et al., 2018)
GO	Electrochemistry	NA	5 pM-5nM	(Lanche et al., 2018)

Note: LOD: limit of detection, NA: No available data.

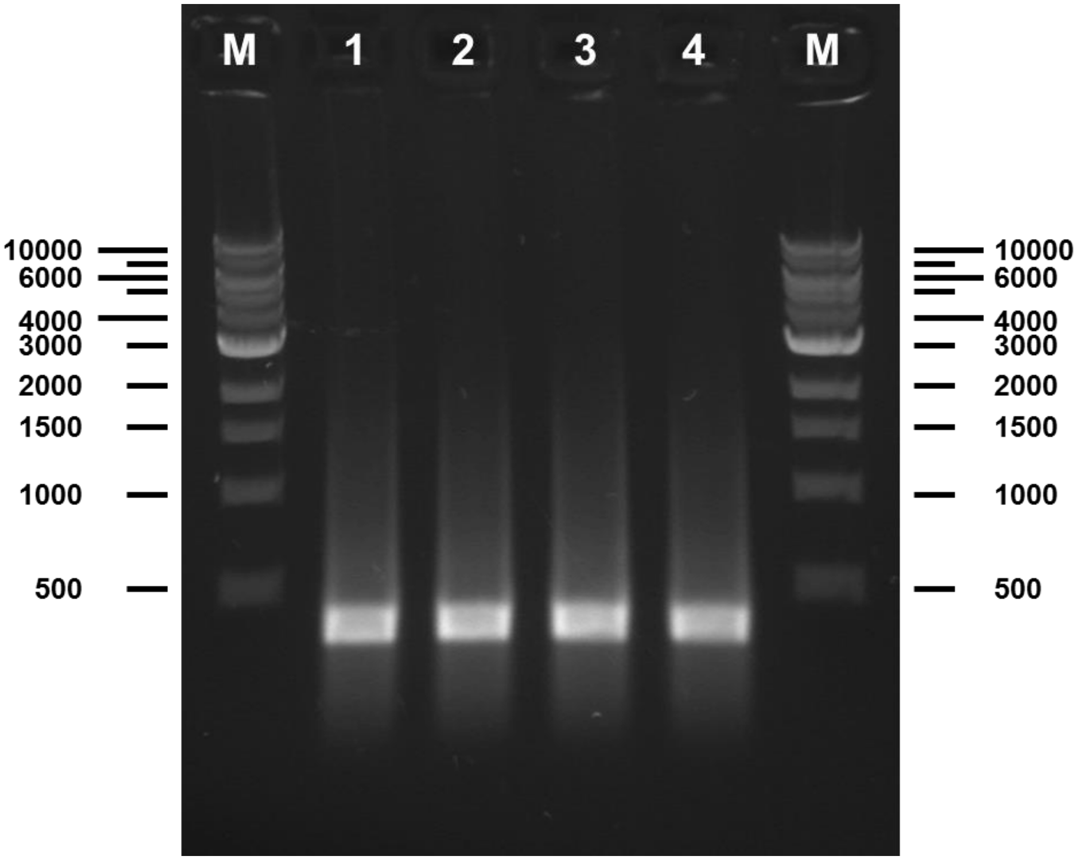

Figure S6. Agarose gel electrophoresis image of the PCR products. Lane 1-2: mutant-type samples; Lane 3-4: wild-type samples. Lane M is DNA ladder. The bright bands were the PCR products near the position of 500 base-pairs DNA markers, consistent with the predicted length of 359 base-pairs for target PCR amplicons. The detailed sequences of the PCR products were confirmed through DNA sequencing.

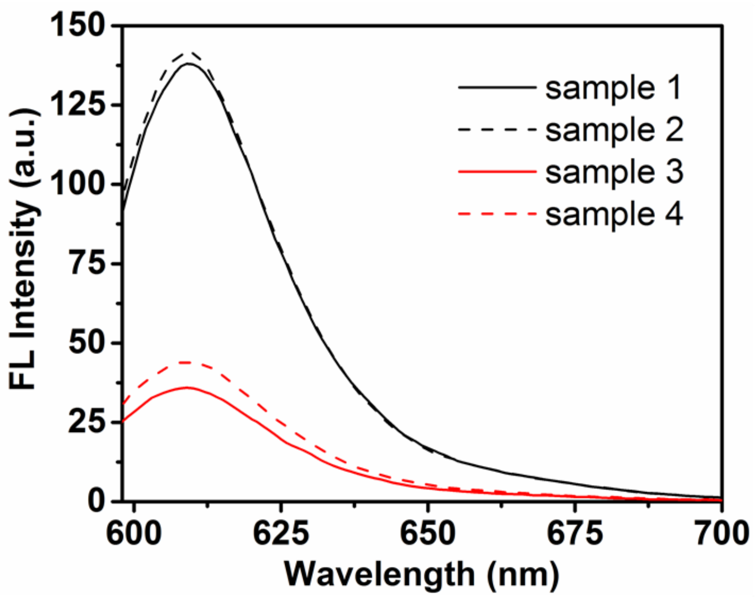

Figure S7. Fluorescence spectra of the human genomic samples (sample 1-2: mutant-type samples; sample 3-4: wild-type samples; P = 1 μM; Exo III = 0.25 U μL^{-1}; Ta_2NiS_5 = 5 μg mL^{-1}).

Reference

Fang, Y., Yang, X., Chen, T., Xu, G., Liu, M., Liu, J., et al. (2018). Two-dimensional titanium carbide (MXene)-based solid-state electrochemiluminescent sensor for label-free single-nucleotide mismatch discrimination in human urine. *Sensor Actuat B-Chem*. 263, 400-407. doi: 10.1016/j.snb.2018.02.102.

Han, Z., Jiang, Q., Zhang, T., Wu, X., Ma, R., Wang, J., et al. (2015). Analyzing large-scale samples confirms the association between the rs1051730 polymorphism and lung cancer susceptibility. *Sci rep*. 5, 15642. doi: 10.1038/srep15642.

Lanche, R., Pachauri, V., Munief, W.-M., Müller, A., Schwartz, M., Wagner, P., et al. (2018). Graphite oxide electrical sensors are able to distinguish single nucleotide polymorphisms in physiological buffers. *FlatChem*. 7, 1-9. doi: 10.1016/j.flatc.2017.12.001.

Xing, S., Xu, X., Fu, P., Xu, M., Gao, T., Zhang, X., et al. (2019). Colorimetric detection of single base-pair mismatches based on the interactions of PNA and PNA/DNA complexes with unmodified gold nanoparticles. *Colloid Surface B*. 181, 333-340. doi: 10.1016/j.colsurfb.2019.05.069.

Xu, W., Xing, S., Xu, X., Xu, M., Fu, P., Gao, T., et al. (2018). Peptide nucleic acid-assisted label-free detection of single-nucleotide polymorphisms based on light scattering of carbon nanotubes. *ACS Omega*. 3, 17835-17841. doi: 10.1021/acsomega.8b02655.

Yan, L., Deng, Z., Shi, H., Xie, B., and Gao, L. (2019). A Method for SNPs Detection Using MoS2@AuNPs and SYBR Green I Combining with Enzyme Digestion. *New J Chem*. doi: 10.1039/C9NJ04319E.
